# Endoscopic Ultrasound-Guided Tissue Acquisition of Pancreaticobiliary Cancer Aiming for a Comprehensive Genome Profile

**DOI:** 10.3390/diagnostics13071275

**Published:** 2023-03-28

**Authors:** Susumu Hijioka, Yoshikuni Nagashio, Yuta Maruki, Yuki Kawasaki, Kotaro Takeshita, Chigusa Morizane, Takuji Okusaka

**Affiliations:** Department of Hepatobiliary and Pancreatic Oncology, National Cancer Center Hospital, Tokyo 104-0045, Japan

**Keywords:** comprehensive genome profile, endoscopic ultrasound-guided tissue acquisition, Oncoguide NCC Oncopanel System, Foundation One CDx cancer genome profile, pancreatic cancer, precision medicine

## Abstract

In recent years, cancer genomic medicine centered on comprehensive genome profile (CGP) analysis has become widely used in the field of pancreatic cancer. Endoscopic ultrasound-guided tissue acquisition (EUS-TA) has played an important role in pancreatic cancer, and recently, more EUS-TA tissue samples are considered for CGP analysis. Differences exist between the Oncoguide NCC Oncopanel System and Foundation One CDx Cancer Genome Profile, which are CGP tests approved by insurance programs in Japan, including the analysis criteria, optimal needle selection for meeting these criteria, and puncture target. It is important to understand not only the specimen collection factors, but also the specimen processing factors that can increase the success rate of CGP testing. Furthermore, cancer genome medicine is expected to enter an era of increasing turbulence in the future, and endoscopists need to respond flexibly to these changes. Herein, we review the current status of cancer genome medicine in pancreatic and biliary tract cancers and cancer gene panel testing using EUS-TA.

## 1. Introduction

Genetic mutations, which are the main cause of cancer, vary among individual patients, even those with the same cancer type, considerably affecting the response to anticancer drugs and prognosis. With the recent expansion and improvement in next generation sequencing (NGS), it has become possible to comprehensively analyze genetic alterations in many cancer-related genes, simultaneously, from tens to hundreds of ng of DNA extracted from a small portion of a tumor specimen, and to identify the many genetic abnormalities that accumulate in cancer. Furthermore, precision medicine for cancer treatment, centered on cancer gene panel testing, began to flourish around 2015. In Japan, comprehensive genome profile (CGP) testing has been covered by insurance programs since 2019, and in the years since, CGP testing has widely penetrated clinical practice.

Pancreatic and biliary tract cancers have an extremely poor prognosis, and are an area where genomic medicine based on the CGP test could be provided. On the other hand, pancreatic and biliary tract cancers are located in organs that are difficult to collect tissue sampling from.

Recently, endoscopic ultrasound (EUS)-guided tissue actuation (EUS-TA) has been commonly used for tissue collection in pancreatic and biliary tract cancers. EUS-TA has recently become important not only in pathological diagnosis, but also in genomic medicine, which enables personalized medicine by performing multiple gene analyses simultaneously. Comprehensive genomic profiling is currently applied in the clinical setting and there is an increasing need for gene analysis using EUS-TA samples.

Thus, in this review, we report the current status of cancer genome medicine in pancreatic and biliary tract cancers and cancer gene panel testing using EUS-TA.

## 2. Comprehensive Genome Profiling of Pancreatic Cancers

The detection of genetic abnormalities and/or the tumor mutation burden on panel tests may lead to opportunities in precision medicine for treatments covered by insurance programs, as well as further clinical trials. Unfortunately, the percentage of patients able to receive treatment for abnormalities found as a result of panel testing is not large, at <15% [1], and the percentage in pancreatic ductal adenocarcinoma (PDAC) is likely even lower. However, as pancreaticobiliary cancer has a very poor prognosis, many patients request CGP testing to attempt even the slightest possibility of receiving a new treatment. In the breakdown of CGP testing in Japan, pancreatic and biliary tract cancers were found to be the second and third most frequent cancer types, respectively, after colorectal cancer [2].

Although mutated *KRAS* is the major oncogenic driver in PDAC and is an attractive treatment target, no effective treatments have been identified for patients with *KRAS*-mutant PDAC, with the exception of the very rare *KRAS G12C* mutation. Sotorasib, which targets the *KRAS G12C* mutation, has shown an anticancer activity in patients with *KRAS G12C* mutated non-small cell lung cancer. The *KRAS G12C* mutation is also found in a small fraction of pancreatic cancers, and sotorasib has shown efficacy in pancreatic cancer in a phase I study.

Other recent studies have indicated that KRAS-wild type pancreatic cancer has various targetable alterations [3,4,5]. Additionally, homologous recombination deficiency (HRD) is common genomic abnormality. *BRCA1* and *BRCA2* are key proteins involved in homologous recombination (HR) and play important roles in DNA double-strand break repair. Hence, PDAC with HRD is reportedly more sensitive to platinum-based chemotherapy, which induces a DNA-damaging effect [6]. Other HR-related genes include *PALB2*, *ATM*, and *ATR*. A meta-analysis reported the pooled prevalence of HR-related gene mutations (germline and somatic mutations) in PDAC as follows: *BRCA1*, 0.9%; *BRCA2*, 3.5%; *PALB2*, 0.2%; *ATM*, 2.2%; and *ATM*, 0.2% [7]. Maintenance therapy with olaparib, a PARP inhibitor, demonstrated efficacy for platinum-sensitive PDAC with *BRCA* pathological variants in a phase III study (the POLO trial) [8]. In addition, pembrolizumab, developed for microsatellite instability-high solid tumors [9], and entrectinib, developed for *NTRK* fusion gene mutations across multiple tumor types [10], have also shown efficacy for pancreaticobiliary cancers.

In the Know Your Tumor^®^ Project study conducted in the United States, 26% of patients with pancreatic cancer underwent CGP testing and had actionable mutations useful for treatment selection. Additionally, the overall survival of patients who received a drug that matched the actionable mutation was 31 months, which was significantly superior to 18.1 months in the control group (hazard ratio: 0.42, *p* < 0.01) [11].

As *KRAS* mutations are found in approximately 95% of cases of pancreatic cancer, it is considered that matched therapy (therapy that matches the genetic abnormality) is indicated in a small number of cases, but these results suggest that NGS testing to find actionable mutations should be performed early in the treatment process. Based on this information, the 2019 National Comprehensive Cancer Network (NCCN) clinical guideline recommends germline testing, tumor/somatic gene profiling, and microsatellite instability/mismatch repair analysis in patients with PDAC [12].

## 3. Comprehensive Genome Profiling of Bile Duct Cancers

Whole-genome sequencing studies in biliary tract cancer have revealed genomic alterations in several oncogenes and tumor suppressor genes, mainly in *KRAS*, *TP53*, *CDKN2A*, and *SMAD4* [13,14,15]. Nakamura et al. revealed that several genetic mutations commonly occur in biliary tract cancers, while others occur according to the cancer site (e.g., intrahepatic/extrahepatic bile duct and gallbladder), in a comprehensive genetic analysis, including a whole-transcriptome sequence analysis [13]. According to their report, common mutations in biliary tract cancers, including gallbladder cancer, were detected in *TP53*, *BRCA1*, *BRCA2*, and *PIK3CA*; intrahepatic and extrahepatic duct-shared mutations were detected in *KRAS*, *SMAD4*, *ARID1A*, and *GNAS*; intrahepatic duct-specific mutations were detected in *FGFR2* fusion, *IDH1/2, EPHA2*, and *BAP1* genes; and extrahepatic duct-specific mutations were detected in *PRKACA/PRKACB* fusion, *ELF3*, and *ARID1B* genes. Jain et al. [16] reviewed the molecular profiles of bile duct cancers and indicated the frequencies of several major gene mutations, as follows: *TP53*, 44–28% in Asians and 6% in Caucasians; *KRAS*, 12–17%; *SMAD4*, 6–20%; *CDKN2A*, 4–5%; *BRCA1/2*, 3–5%; and *PIK3CA*, 3–5%. FGFR2 inhibitors Pemigatinib [17] and Fucibatinib [18] have been shown to be effective in cases of FGFR2 fusion gene abnormalities. Pemigatinib showed a favorable response rate of 35.5% (95% CI: 26.5–45.4) in a global phase II clinical trial (FIGHT-202 study) [17]. Fucibatinib also showed a good response rate of 42% (95% confidence interval, 32 to 52).

In the PRELUDE study [19], which investigated the expression rate of the FGFR2 fusion gene in biliary tract cancers in Japan, the FGFR2 fusion gene was found in 7.4% (20/272) of intrahepatic cholangiocarcinoma and 3.6% (3/83) of hilar cholangiocarcinoma, and was associated with young age (≤65 years: *p* = 0.070) and HCV/HBV infection (*p* = 0.037). In addition, ivosidenib, an IDH1 inhibitor, is available for the treatment of IDH1 mutations, which are considered positive in 10–20% of intrahepatic cholangiocarcinoma.

A phase III comparative study (ClarIDHy study) [20], ivosidenib significantly prolonged progression-free survival versus placebo (median 2.7 vs. 1.4 months, HR 0.37, 95%).

In the 5th edition of the WHO classification revised in 2019, it classified intrahepatic cholangiocarcinoma into two types: large duct type and small duct type.

This classification is reported to reflect the findings of both types, as well as pathological features and molecular profile.

In terms of the molecular profile, the large duct type has *KRAS* mutations and *SMAD4* deletions are more frequent, whereas the small duct type has a higher frequency of *IDH1* mutation and *FGFR2* fusion abnormality.

The recognition of these two categories based on imaging findings and pathological features will help to improve the efficiency of the diagnosis and treatment of small ductal carcinoma.

In addition, in biliary tract cancer, there have been other responses such as dabrafenib + trametinib (response efficiency 47%) in *BRAF V600E* (response rate 47%) and pertuzumab + trastuzumab (response rate 23%) in HER2-positive patients (response rate 23%).

The development of niraparib for *BRCA1/2* mutations and olaparib for patients with other DNA repair gene mutations is also underway. There are many promising target-drug combinations for treating biliary tract cancer.

## 4. Comprehensive Genome Profiling of across Tumor Types

Drugs targeting rare alterations found in different solid tumors, such as microsatellite instability-high (MSI-H), a high tumor mutation burden (TMB-H), and NTRK fusions, have obtained approval across tumor types [21]. Tumor MSI status and TMB have been shown to play a significant role in the therapeutic efficacy of immune checkpoint inhibitors [22]. The Food and Drug Administration approved pembrolizumab, an antibody against the programmed cell death-1 (PD-1) protein, for the treatment of patients with MSI-H solid tumors in 2017 and TMB-H solid tumors in 2020 [23], but it is limited to a small number of cases [22]. In pancreatic cancer, the frequency of MSI-H is as low as 2.5% [24]. The rate of MSI-H biliary cancer is reported to be 1–3% [25,26].

## 5. Results of Genomic Medicine Using EUS-TA in Pancreaticobiliary Cancers

Currently, two types of CGP tests for tumor-tissue specimens are covered by insurance in Japan: the OncoguideTM NCC OncoPanel System (NCCOP) and the Foundation One CDx Cancer Genome Profile (F-One). To perform precision medicine using EUS-TA specimens, it is necessary to be aware of the differences between these two panel tests (Table 1).

The major clinical differences between NCCOP and F-One are the presence/absence of an analysis of germline mutations and the number of genes analyzed. Furthermore, a major difference of importance to endoscopists is the difference in criteria regarding the amount of tissue required (tissue section area and recommended tumor cell content). As shown in Table 1, F-One requires a larger tumor cell area than NCCOP. Although F-One searches for a large number of genes, it also requires a larger amount of tissue (>25 mm^2^), which is a limitation of EUS-TA. In contrast, NCCOP only requires a tissue area of >4 mm^2^, and small EUS-TA specimens are more easily submitted.

Recently, liquid biopsy testing of blood and other body-fluid specimens has been developed as a new analytic method [27,28,29], and the F-One Liquid CDx Cancer Genome Profile [30] test for blood specimens is now covered by insurance programs in Japan. Although the liquid biopsy test is very useful for pancreaticobiliary cancers, for which the collection of samples for genetic testing is difficult, its sensitivity for detecting *KRAS* abnormalities is lower than that of the test using tumor tissue specimens [31], and tumor tissue specimens are superior in terms of certainty. The FGFR2 fusion gene is the most common genetic abnormality found in intrahepatic cholangiocarcinoma, and its detection using liquid biopsy is low. Therefore, in pancreatic and biliary tract cancers, it is still desirable to submit specimens from tissues as much as possible in order to identify the gene abnormality that is the target of treatment.

Table 2 summarizes the differences between the tumor and liquid tests. For patients with a promising prognosis, cancer genomic testing using tumor tissue samples is the first choice.

For CGP testing, DNA is extracted from a formalin-fixed paraffin-embedded (FFPE) specimen of the tumor and then analyzed. In recurrent cases, resected specimens can be used as an FFPE sample (in principle, within 3 years); however, for unresectable PDAC, CGP testing is usually performed on tissue samples obtained by EUS-TA or percutaneous liver biopsy in cases with liver metastases. Although a liver biopsy often provides a sufficient amount of the tumor for the panel test [32], in patients with locally advanced PDAC, lung metastases, peritoneal dissemination only, or in whom a liver biopsy is difficult to perform, tissue samples from EUS-TA are needed. However, PDAC is representative of low-cellularity tumors with abundant stromal components, and the tumor cell content is reported to be 5–20% [33], rendering CGP testing a relatively difficult task. Furthermore, EUS-TA specimens also contain various contaminants (e.g., gastrointestinal epithelium and stroma), which certainly degrade the quality of the specimen for genome analysis. Thus, the feasibility of CGP testing using EUS-TA specimens has attracted much interest.

In recent years, several studies have reported on CGP analysis using FFPE samples obtained by EUS-TA in pancreatic tumors [32,34,35,36,37]; these studies are summarized in Table 3. The success rates of the panel tests varied markedly, ranging 39.2–100%. This is due to the different types of panel tests used and the lack of uniform standards for the tumor-containing cell rate, tissue area, and DNA content required for the tests.

The amount of input DNA required for NGS depends on the platform, ranging ~10–300 ng. It is estimated that approximately 2000 tumor cells are needed to obtain 10 ng of DNA [38]. Regarding the amount of DNA extracted, Park et al. reported that the mean extracted DNA amounts in the NGS success and failure groups were 540 ng and 142 ng, respectively, and the success rate of the NGS analysis was improved from 57.4% (109/190) to 76.2% (109/143) when the amount of extracted DNA was more than 50 ng [37].

Hence, a sufficient amount and concentration of extracted DNA improves the success rate of the NGS analysis.

Only one study (number of cases > 10) on CGP testing using EUS-TA for biliary tract cancer has been reported. This is because compared with pancreatic lesions, bile duct lesions are more difficult to puncture, and there are fewer conditions in which they can be punctured. Hirata et al. [39] performed cancer gene panel testing for 21 cases of biliary tract cancer using EUS-TA samples, with 50 cancer genes being the analysis target. This retrospective study achieved deep sequencing coverage and identified pathogenic alterations in 95.2% (20/21) of the patients with biliary tract cancer using EUS fine-needle aspiration (EUS-FNA) samples. However, the analysis target of 50 cancer genes was small compared with the usual number in CGP testing, which may have been the reason for the good results. However, EUS-TA may be useful for CGP testing in patients with suspected biliary tract cancer, as in PDAC.

## 6. Tips for Improving the Success Rate of CGP Analysis with EUS-TA in Pancreaticobiliary Tract Cancers

**Figure 1:**
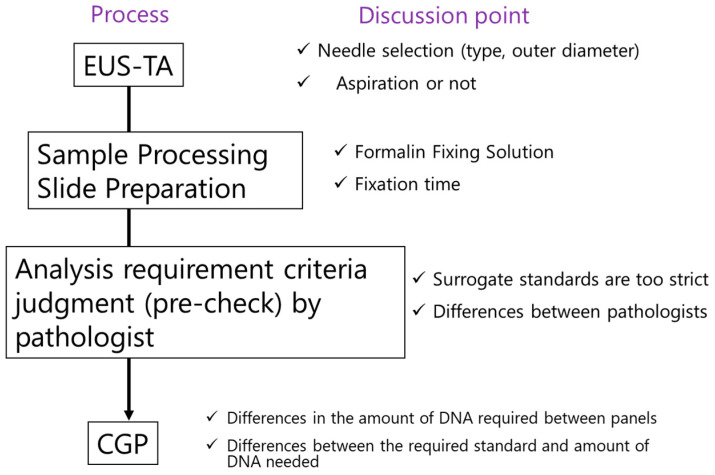
Process from EUS-TA to CGP analysis and discussion points. CGP: comprehensive genome profile; EUS-TA: endoscopic ultrasound-guided tissue acquisition.

Figure 1 shows the flow of the gene panel testing using EUS-TA. From EUS-TA to CGP testing, there are five main processes, each of which is important to obtain a high success rate: (1) EUS-TA, (2) specimen processing and formalin fixation, (3) slide preparation, (4) pre-check by pathologist, and (5) submission to CGP. The factors affecting these processes are discussed below.

### 6.1. Selection of Needle Type/Size for EUS-TA

Various preanalytical factors, including tumor cellularity, tumor fraction, and tumor viability, are associated with DNA quality; therefore, specimen collection and processing methods, including the selected needle type and size, are important for this reason.

In recent years, puncture needles with a unique needle tip shape, known as fine needle biopsy (FNB) needles, have emerged to improve the tissue specimen volume. Several types of needles are available, including Francine and fork-tip needles. These needles are similar to conventional FNA needles in that they use the puncture aspiration method, but they differ in tip shape, which increases the amount of tissue sampled. The difference in the amount of tissue sampled with FNA and FNB needles has been verified in several publications, and it has been reported that the FNB needle has a significantly higher success rate when CGP analysis is the primary objective [35,40,41,42]. Based on these results, the FNB needle is now recommended for EUS-TA considering CGP [43,44,45]. In addition, according to a recent network meta-analysis, Francine and fork-tip needles, among the FNB needles, show the best performance for tissue collection, and it is recommended that one of these two types be selected for CGP purposes as well [46].

Regarding needle size, Park et al. compared 19-G/22-G needles to 25-G needles and found [37] that a 19-G/22-G diameter needle was more appropriate for NGS (success rate: 63.2% vs. 38.8%, *p* = 0.003). Furthermore, in a randomized controlled trial of 25-G FNA and 19-G/22-G FNB needles, the percentage of patients who fulfilled F-One analysis criteria with a single puncture was greater with 19-G/22-G FNB needles (78%) compared with 25-G FNA needles (14%) [40]. Thus, the success rate of CGP analysis is low for 25-G needles. EUS-FNB and needle size are considered predictors of successful NGS in PDAC. Several studies comparing the adequate tissue rate of NGS analysis in PDAC between EUS-FNA and EUS-FNB have found that EUS-FNB is more suitable for NGS analysis (EUS-FNA: 14–66.9% vs. EUS-FNB: 70.4–90.9%) [32,35,40]. According to Kindel et al., the mean DNA concentrations in PDAC by FNB and FNA needles were 5.930 (standard deviation [SD] 0.881) µg/mL vs. 3.365 (SD 0.788) µg/mL, respectively (*p* = 0.01). Significantly more DNA was obtained using the FNB needle. Furthermore, the success rate of NGS analysis in PDAC is better with 19-G or 22-G than with 25-G needle (odds ratio [OR] 2.19, 95% confidence interval [CI]: 1.08–4.47) [37]. There are five types of suction methods for EUS-TA: “no suction”, “slow-pull technique”, “dry suction”, “modified wet suction”, and “wet suction.” According to a recent network meta-analysis [47], the “no suction” technique was significantly inferior to the other techniques. Consequently, “modified wet suction” resulted as the best technique (SUCRA 0.90). Based on these results, “modified wet suction” might be recommended for NGS.

**Figure 2:**
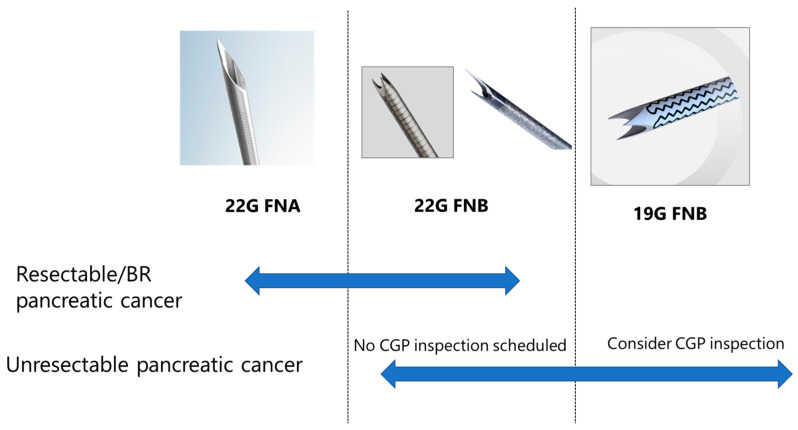
EUS-TA recommendations. For resectable/borderline resectable (BR) pancreatic cancer, 22-G FNB is the first choice because the main focus is on a benign/malignant diagnosis; however, 22-G FNA is selected in cases of difficult puncture. For unresectable pancreatic cancer and when CGP analysis is considered, 19-G FNB is the first choice; if CGP analysis is not planned, 22-G FNB is selected based on puncture performance and safety. CGP: comprehensive genome profile.

Ikeda et al. [48] retrospectively investigated the percentage of cases achieving NCCOP analysis criteria (153 cases in total; FNB 19-G needle: 75 cases, FNB 22-G needle: 43 cases, and FNA 22-G needle: 35 cases). Overall, only 39.0% (60/153) of cases met the NCCOP analysis criteria. The percentage meeting criteria was significantly higher with 19-G FNB needles (56.0% [42/75]) than with other needles (22-G FNB: 32.6% [14/43] and 22-G FNA: 11.4% [4/35]) (*p* < 0.01). Furthermore, FNB (OR = 3.91, 95% CI: 1.17–13.0, *p* = 0.0267) and 19-G needles (OR = 2.44, 95% CI: 1.12–5.30), *p* = 0.0247) were independent factors contributing to the achievement of the NCCOP analysis criteria. Based on these results, the 19-G FNB needle is the first choice for unresectable (UR) pancreatic cancer in our institution for cases with CGP analysis in mind. However, as the needle size becomes larger, puncturing becomes more difficult and the risk of complications increases; thus, 22-G FNB (or FNA) is used for UR pancreatic cancer and resectable/borderline resectable pancreatic cancer that is not scheduled for CGP analysis (Figure 2).

A recent study indicated that the percentage of patients who achieved genomic criteria in EUS-TA specimens of primary pancreatic cancer (37.1% [53/143]) was significantly lower than the percentage of patients who achieved genomic criteria in EUS-TA specimens from metastatic sites (liver metastases or lymph nodes) (70% [7/10]) (*p* = 0.049). Thus, collecting samples from liver metastases for EUS-TA in anticipation of CGP testing may be recommended.

A prospective study [49] with a primary endpoint of the percentage of specimens obtained by EUS-TA using a 19-G FNB needle achieving NCCOP analysis criteria reported that the proportion of patients meeting the predefined criteria to be considered valid was 63.6% (95% CI: 47.22–80.05). The median tumor cell content was 60% (range: 7.5–85). Tissue sizes were ≤4 mm^2^ and 4–16 mm^2^ in 10 (30.3%) and 23 (69.7%) patients, respectively. Moreover, none of the patients met the F-One analysis suitability criteria. The samples were actually submitted to NCCOP analysis in 36.4% (12/33) of cases, of which 100% (12/12) were evaluable. The mean DNA content was 1050.7 ng (SD 684.2). Currently, there is no clear indication regarding the appropriate number of punctures for CGP. However, it has been suggested that the use of macroscopic on-site evaluation (MOSE) may contribute to a reduction in the number of EUS-TA punctures [50], and this may be applied to CGP as well.

There are also some important considerations regarding pre-analytical specimen-processing methods. FFPE DNA extraction methods may cause DNA fragmentation and chemical modification. Therefore, the formalin fixation time should be shortened for small specimens, such as EUS-FNA specimens. In addition, the quality of nucleic acids deteriorates with time and the most recent specimen is the most suitable for NGS.

### 6.2. Specimen Processing and Formalin Fixation

Although the optimal specimen-processing method for EUS-TA with CGP testing has not yet been established, the following three points are important in EUS-TA. (1) Tissues collected from biopsies should be promptly immersed in a fixative solution for fixation. (2) A 10% neutral buffered formalin solution should be used in the composition of the formalin fixative. (3) Formalin fixation should be performed for 6–48 h [51].

In real-world practice, if EUS-TA is performed before a long holiday, over fixation of formalin is a concern, which is especially relevant to point out (3). Therefore, it is preferable to avoid performing EUS-TA before a long holiday, if possible, when considering CGP analysis.

### 6.3. Pathologist’s Criteria for Genome Analysis (Pre-Check)

The pathologist’s determination of whether the required criteria for analysis have been met (commonly called the pre-check) is based on the tumor cell content and tissue section area. This pre-check plays a gatekeeping role, before submission to the actual CGP examination. This is because CGP testing is very expensive; if CGP testing cannot be performed due to an insufficient specimen volume, the patient burden will be high. Additionally, from the standpoint of medical resource use, the pre-check should be strictly required in terms of testing conditions. Thus, the pre-check may be extremely strict.

Additionally, specific criteria for EUS-TA are lacking. The current pre-check based on the tissue area and tumor content does not conform to the criteria for EUS-TA specimens, rendering it difficult to make a judgment. Thus, the pre-check may need to be revised in the future with criteria specifically for EUS-TA. This issue should be clarified in a multicenter prospective study.

## 7. Conclusions

We described genomic diagnosing using EUS-TA in pancreatic cancers. In the past, EUS-FNA was primarily used for diagnosis; however, EUS-FNB, which can collect a larger amount of tissue, has become the mainstream specimen collection method, as a larger amount of tissue is required for EUS-TA in the current era of genomic medicine. Although the amount of DNA required for panel testing is expected to decrease as genomic medicine advances in the future, endoscopists still face numerous demands at this time. To respond to these demands and contribute to the patient’s cancer treatment to the best extent, it is necessary for endoscopists to make continuous efforts to study and collect information on a daily basis.

## 8. Future Directions

CGP will become more advanced and require less tissue. It is also expected that CGP testing will become possible with smaller amounts of DNA, such as with the development of gene panel tests specific for biliopancreatic cancer. At this point, however, it is important to utilize many EUS-TA techniques and devices to improve the success rate of CGP testing with EUS-TA.

## Figures and Tables

**Table 1:** Differences between two cancer gene panel tests.

Test Name	NCC Onco Panel	Foundation One CDx
Number of genes analyzed	114 genetic variants 12 fusion genes	324 gene mutations 36 fusion genes
Determination of microsatellite instability	No	Yes
Determination of tumor mutation burden	Yes	Yes
Analysis of germline mutations	Possible	Impossible
Specimen used	Tumor specimen (FFPE) Normal (peripheral blood)	Tumor specimen (FFPE)
Required area (tissue section area and number of slides required)	In principle, 16 mm^2^ or more (4 mm^2^ is acceptable) 5–10 µm sheets	25 mm^2^ or more 5–10 µm sheets
Specimen eligibility criteria (recommended tumor cell content)	Tumor cell content ≥ 20% DNA input amount: 200 ng	Tumor cell content ≥ 20% (Optimal 30% or more)

FFPE: formalin-fixed paraffin-embedded.

**Table 2:** Differences between EUS-TA and liquid biopsy panel analyses.

	EUS-TA (Tissue)	Liquid Biopsy (Plasma)
Invasiveness	Yes	Low
Turnaround time	Long (7–8 weeks from the time of tissue collection)	As short as 3–4 weeks
KRAS mutation detection rate	83–88%	Approximately 60%
Other advantages	Applications in RNA, whole exon, and whole genome analyses	Overcomes the problem of intra-tumor heterogeneity Application in the early detection of small residual lesions
Disadvantages	Difficulty in specimen collection, risk of complications, specimen degradation over time, and heterogeneity issues within tumors	Results depend on the timing of specimen collection, tumor cell content cannot be evaluated in advance, limited sensitivity in detecting fusion genes and copy number, and uncertainty in determining TMB and MSI.

EUS-TA: endoscopic ultrasound-guided tissue acquisition; MSI: microsatellite instability; TMB: tumor mutation burden.

**Table 3:** Success rate of cancer genomic testing using EUS-TA FFPE for pancreatic tumors.

First Author	Design	*n*	Type of Tumor	Needle Size/Type	Types of Gene Panel Tests	Minimum Amount of DNA Required	Requirement	Percentage Meeting NGS Analysis Criteria/Successful Rate	DNA Amount DNA Concentration	Judgment Method	Frequency of Genomic Alternations (PDAC)
Young G (2013)	Retrospective	23	Adenoca. NEC	N/A	Custom panel (287 genes)	50 ng	Tumor cell content ≥ 20%	100% (23/23)	N/A	Actual Panel Inspection	KRAS 83% (15/18) CDKN2A 44% (8/18)
Gleeson FC (2016)	Retrospective	47	Adenoca. papillary carcinoma	N/A	Human Comprehensive Cancer GeneRead DNAseq Targeted Panel V2 (160 genes)	DNA concentration: 5 ng/μL	Tumor cell content ≥ 20%	61.7% (29/47)	66.9 ng/μL (range 9.3–164)	Actual Panel Inspection	KRAS 93.1% (27/29) TP53 72.4% (21/29) SMAD4 31% (9/29) GNAS 10.3% (3/29)
Elhanafi S (2018)	Retrospective	167	Adenoca.	22G FNA (*n* = 145) 22G FNB (*n* = 22)	TruSeq Amplicon Cancer Panel (47 genes)	N/A	Tumor cell content ≥ 10%	70.10% 22G FNB (90.9%) 22G FNA (66.9%)	N/A	Analysis Criteria (Pre-check)	KRAS 88% (22/25) TP53 68% (17/25) SMAD4 16% (4/25)
Larson BK (2018)	Retrospective	76	Adenoca. Acinar cell carcinoma	EUS-FNA (*n* = 7) EUS-FNB (*n* = 54) 19–25G Others (*n* = 15)	FoundationOne (324 genes)	N/A	Tissue slicing area ≥ 25 mm^2^ Tumor cell content ≥ 20%	EUS-FNA 42.9% EUS-FNB 70.4%	N/A	Analysis Criteria (Pre-check)	N/A
Park JK (2020)	Retrospective	190	Adenoca.	EUS-FNB 19–25G	Cancer SCANTM v1 (83 genes)	50 ng	N/A	57.4% (109/190)	NGS success: 1.42 ± 1.57 μg NGS failure: 0.54 ± 1.70 μg	Actual Panel Inspection	KRAS 78.9% (86/109) TP53 60.6% (66/109) SMAD4 30.3% (33/109) CKDN2A 25.7% (28/109)
Ishizawa T (2020)	Retrospective	26	Adenoca.	EZ shot 2 22G ProCore 22G	AmpliSeq Comprehensive Cancer Panel (409 genes)	N/A	N/A	100% (26/26)	mean 171 ng (range 34–478)	Actual Panel Inspection	KRAS 92% (24/26) TP53 50% (13/26) SMAD4 31% (8/26) CDKN2A 15% (4/26)
Kandel P (2021)	Prospective	50	Adenoca. NET	FNA 25G FNB 22G or 19G	FoundationOne (324 genes)	10 ng	Tissue slicing area ≥ 25 mm^2^ Tumor cell content ≥ 20%	25G FNA (14%) FNB 22G/19G (78%)	EUS-FNA: mean 3.365 ng/μL EUS-FNB: mean 5.930 ng/μL	Analysis Criteria (Pre-check)	N/A
Carrara S (2021)	Prospective	33	Adenoca.	Acquire 22G	AmpliSeq Comprehensive Panel v3 161 genes	DNA concentration: 3.3 ng/μL	N/A	97.0% (32/33)	N/A	Actual Panel Inspection	KRAS 94% (30/32) TP53 78% (25/32) SMAD4 13% (4/32) CDKN2A 9% (3/32) GNAS 9% (3/32)
Habib JR (2021)	Retrospective	56	Adenoca.	N/A	Ampliseq Custom Panel 9 genes	DNA concentration: 3.3 ng/μL	N/A	100% (56/56)	N/A	Actual Panel Inspection	KRAS 85.7% (48/56) TP53 32.1% (18/56) SMAD4 3.6% (2/56) CKDN2A 3.6% (2/56)
Ikeda G (2022)	Retrospective	153	Adenoca.	19G FNB (*n* = 75) 22G FNB (*n* = 43) 22G FNA (*n* = 35)	NCC Oncopanel (126 genes)	200 ng	Tissue slicing area ≥ 5 mm^2^ Tumor cell content ≥ 20%	39.0% (60/153 cases) 19G FNB (56%) 22G FNB (32.6%) 22G FNA (11.4%)	19G FNB1062.1 ng 22G FNB 411.32 ng	Analysis Criteria (Pre-check)	KRAS 93.3% (28/30) TP53 76.7% (23/30) SMAD4 30.0% (9/30) BRCA 10% (3/30)
Hisada (2022)	Prospective	33	Adenoca.	19G FNB (Top Gain)	NCC Oncopanel (126 genes)	200 ng	Tissue slicing area ≥ 5 mm^2^ Tumor cell content ≥ 20%	63.6% (12/33)	N/A	Analysis Criteria (Pre-check)	KRAS 100% (12/12) TP53 66.7% (8/12) SMAD4 66.7% (8/12) GNAS 33.3% (4/12) BRCA 25% (3/12)

Adenoca.: adenocarcinoma; EUS-TA: endoscopic ultrasound-guided tissue acquisition; FFPE: formalin-fixed paraffin-embedded; FNA: fine needle aspiration; FNB: fine needle biopsy; NEC: neuroendocrine carcinomas; NET: neuroendocrine tumor; NGS: next generation sequencing; PDAC: pancreatic ductal adenocarcinoma.

## Data Availability

No new data were created or analyzed in this study. Data sharing is not applicable to this article.
